# Altitude-Related Adaptation in Freshwater Snails (*Cipangopaludina cathayensis*): Insights from Biochemical, Transcriptomic, and Metabolomic Analyses

**DOI:** 10.3390/ani16152440

**Published:** 2026-08-06

**Authors:** Yuhan Jiang, Qigen Liu, Qing Liu, Weijun Wu, Jiamin Sun

**Affiliations:** 1 Research Centre of Environmental Ecology and Fish Nutrition, Certificated by the Ministry of Agriculture and Rural Affairs, College of Fisheries and Life Science, Shanghai Ocean University, Shanghai 201306, China; 2 Key Laboratory of Exploration and Utilization of Aquatic Genetic Resources, Ministry of Education, College of Fisheries and Life Science, Shanghai Ocean University, Shanghai 201306, China; 3 Shanghai Engineering Research Center of Aquaculture, College of Fisheries and Life Science, Shanghai Ocean University, Shanghai 201306, China; 4 Shuoyuan Agricultural Technology Co., Ltd., Lishui 323800, China; 5 Qingyuan County Agriculture and Rural Affairs Bureau, Lishui 323800, China

**Keywords:** *Cipangopaludina cathayensis*, high-altitude, biomineralization, transcriptomic, metabolomic

## Abstract

High-altitude environments expose aquatic organisms to complex environmental challenges, including fluctuations in dissolved oxygen levels, water temperature, and other habitat conditions. The freshwater snail *Cipangopaludina cathayensis* has limited dispersal ability and is strongly associated with local habitat conditions, making it a suitable species for investigating biological responses to long-term environmental variation. In this study, high-altitude and low-altitude populations of *Cipangopaludina cathayensis* were compared using biochemical assays, shell elemental composition analysis, transcriptomics, and metabolomics to explore altitude-associated physiological and molecular responses. Environmental monitoring showed that the high-altitude habitat exhibited lower water temperatures, greater thermal variability, and significantly higher dissolved oxygen availability than the low-altitude habitat, indicating distinct environmental conditions between the two populations. The high-altitude population exhibited significantly higher superoxide dismutase (SOD) and pyruvate kinase (PK) activities than the low-altitude population, whereas malondialdehyde (MDA) content and total ATPase (T-ATPase) activity showed no significant differences between populations. Shell microstructure and elemental analyses revealed altitude-associated differences in biomineralization. Transcriptomic analysis identified differentially expressed genes involved in intracellular transport, including *KIF10*, *KIF11*, *KIF13*, *KIF17*, *MYO3*, and *MYO7A*, as well as apoptosis-associated genes, including *CASP2*, *CASP3*, and *BCL*-*2*. Integrated transcriptomic and metabolomic analyses further highlighted purine metabolism, D-amino acid metabolism, insulin resistance, and alanine, aspartate and glutamate metabolism as shared enriched pathways between the two populations. These findings indicate that *Cipangopaludina cathayensis* may cope with high-altitude environments through coordinated regulation of antioxidant defense, intracellular transport, apoptosis-associated pathways, and metabolic homeostasis.

## Simple Summary

High-altitude environments expose aquatic animals to multiple environmental challenges, including fluctuations in dissolved oxygen levels and water temperature. These environmental conditions can affect the physiology, shell biomineralization, and metabolism of aquatic animals. The freshwater snail (*Cipangopaludina cathayensis*) has limited dispersal ability and is strongly influenced by local habitat conditions, making it a suitable species for investigating how environmental variations affect physiological and molecular responses in freshwater organisms. In the current study, we compared high-altitude and low-altitude populations of *Cipangopaludina cathayensis* using biochemical analyses, shell elemental characterization, transcriptomic analysis, and metabolomic approaches. The high-altitude population exhibited enhanced physiological responses associated with antioxidant defense and energy metabolism, accompanied by differences in shell biomineralization characteristics between the two populations. Integrated multi-omics analyses further revealed coordinated changes in stress-responsive pathways and metabolic regulation. Together, these findings provide new insights into how freshwater snails respond to environmental variation in high-altitude environments.

## 1. Introduction

High-altitude environments pose substantial challenges to living organisms, including hypoxia, large diurnal temperature fluctuations, and intense ultraviolet radiation. These factors impose strong environmental pressures on resident organisms and can induce phenotypic plasticity and adaptive responses, leading to physiological and molecular adjustments to altitude-associated stressors [1,2,3,4]. Aquatic animals may respond to high-altitude environments through coordinated regulation of oxygen utilization, energy metabolism, cellular protection, and structural adaptation. For example, comparative transcriptomic and genomic studies of Tibetan fishes have revealed accelerated evolution and positively selected genes associated mainly with hypoxia response, energy metabolism, blood circulation, protein regulation, and sensory adaptation [5,6]. In addition to metabolic regulation, structural adaptation has also been reported. For instance, genetic variation in the scale-loss candidate gene *Fgfr1a* has been suggested to contribute to dermal skeletal modification in schizothoracine fishes [7]. Previous studies have shown that hypoxic aquatic environments and pronounced diurnal temperature fluctuations in high-altitude aquaculture systems may affect the growth, survival, tissue health, and intestinal microbiota stability of rainbow trout (*Oncorhynchus mykiss*) [8,9]. Similarly, research on high-altitude land snails has shown that altitude-related thermal conditions can influence seasonal activity patterns and metabolic responses [10]. Life-history traits of the land snails (*Arianta arbustorum*) vary along altitudinal gradients, with increasing elevation associated with smaller shell size and delayed maturation [11]. Moreover, variation in adult shell morphology and life-history traits in the land snail (*Oreohelix cooperi*) was strongly associated with mean annual temperature and the highly correlated variable elevation [12]. At the community level, studies along elevational gradients have shown that land-snail diversity, species richness, and niche occupancy generally decline with increasing altitude, indicating that elevational gradients can shape molluscan distribution patterns and community structure [13,14]. These studies provide important ecological evidence that mollusks are responsive to altitude environments. However, the biochemical, structural, transcriptomic, and metabolomic mechanisms underlying altitude-associated physiological and molecular responses in freshwater gastropods remain poorly understood.

Physiological parameters, including superoxide dismutase (SOD), pyruvate kinase (PK), total ATPase (T-ATPase) activities, and malondialdehyde (MDA) content, serve as biomarkers for evaluating antioxidant capacity, energy metabolism, and physiological adaptation to environmental change [10,15]. Transcriptomic, proteomic, and metabolomic approaches have become powerful tools for investigating aquatic organisms at the systems level [16,17]. Therefore, the integration of transcriptomic and metabolomic analyses can help elucidate the molecular and metabolic mechanisms underlying molluscan adaptation to contrasting altitudinal environments. Mollusks, particularly gastropods, generally have limited dispersal ability and a strong dependence on local microhabitat conditions [18]. Their close associations with temperature, humidity, substrate characteristics, and resource availability make them useful model organisms for investigating ecological and evolutionary responses to elevational environments. *Cipangopaludina cathayensis* is a common freshwater mollusk widely distributed in various freshwater habitats, including rice fields, lakes, swamps, rivers, ditches, and paddy fields [19,20,21,22]. The high-altitude population of *C. cathayensis* examined in this study was collected from terraced rice paddies in Hehu Village, Qingyuan County, Zhejiang Province, China, at an elevation of approximately 1029 m, and this population has previously been referred to as *Cipangopaludina hehuensis*. As in other mollusks, the shell of *C. cathayensis* is a biomineralized structure composed predominantly of calcium carbonate, which is commonly deposited as calcite or aragonite within an organic matrix. Elemental analyses of molluscan shells typically identify calcium (Ca), oxygen (O), and carbon (C) as the principal constituents [23,24,25]. Shell formation and shell properties are highly plastic and can be influenced by environmental factors, including temperature, carbonate chemistry, and calcium availability [26,27]. Previous studies have shown that environmental change can alter shell morphology, microstructure, hardness, and thickness in gastropods and other mollusks, indicating that shell traits may reflect habitat-associated adaptive responses [28,29,30]. Long-term exposure to this unique high-altitude habitat may have contributed to distinct morphological characteristics in the high population compared with the low population, particularly its thinner shell. This trait may represent an environmentally induced response associated with local conditions, such as low temperature and limited calcium availability.

In this study, we compared the high-altitude and low-altitude populations of *C. cathayensis* at both physiological and molecular levels. We performed biochemical parameter assays to assess population differences in antioxidant defense and energy metabolism, characterized shell microstructure and elemental composition using scanning electron microscopy and energy-dispersive X-ray spectroscopy (SEM–EDS), and performed an integrated analysis of transcriptomic and metabolomic data to identify critical genes, metabolites, and pathways associated with altitude-related physiological responses. Given the contrasting thermal and dissolved oxygen conditions between the two paddy-field habitats, we hypothesized that *C. cathayensis* populations inhabiting different elevations would exhibit distinct physiological and molecular strategies associated with environmental variation. Specifically, we predicted that differences in thermal regimes and oxygen availability would be reflected in antioxidant capacity, energy metabolism, shell biomineralization, and transcriptomic and metabolomic regulation. By combining physiological, structural, and multi-omics evidence, this study aimed to elucidate the coordinated mechanisms underlying environmental responses in freshwater gastropods and to provide a theoretical basis for future functional studies and conservation management.

## 2. Materials and Methods

### 2.1. Measurement of Water Temperature and Dissolved Oxygen Levels

Water temperature and dissolved oxygen (DO) concentrations were measured daily at two fixed time points (06:00 and 14:00) from 1 to 31 July 2025 at both sampling sites. The measurements recorded at 06:00 were defined as morning values (AM), whereas those recorded at 14:00 were defined as afternoon values (PM). The high-altitude site was located at Shuoyuan Agricultural Technology Co., Ltd. (Lishui, China; 27.78° N, 119.28° E; 1029 m above sea level), whereas the low-altitude site was located at Yuhe Seed Industry Co., Ltd. (Lishui, China; 27.60° N, 118.99° E; 394 m above sea level). Water temperature was recorded using a HOBO MX Temp 400 Data Logger (Onset Computer Corporation, Cape Cod, MA, USA), whereas DO was measured using a HOBO DO Logger U26-001 (Onset Computer Corporation, Cape Cod, MA, USA). At both sites, the data loggers were secured horizontally, with the sensors positioned approximately midway between the sediment surface and the water surface. The instruments remained fully submerged throughout the monitoring period and were installed to avoid direct contact with the sediment, rice stems, water inlets, and drainage outlets.

### 2.2. Snail Management and Sample Collection

Healthy adult female snails were collected from two geographically distinct sites in Qingyuan County, Zhejiang Province, China, on 6 July 2025. The high-altitude population was collected from Shuoyuan Agricultural Technology Co., Ltd., whereas the low-altitude population was collected from Yuhe Seed Industry Co., Ltd. To minimize variation associated with developmental stage, sex, and body size, only size-matched adult female snails were selected. Their mean shell length was 32.11 ± 2.10 mm.

A total of 58 individuals were used, including 29 snails from each population. Within each population, 15 individuals were used for biochemical assays, 8 individuals were used for shell microstructure and elemental composition analyses, and 6 individuals were used for both transcriptomic and metabolomic analyses. The same six individuals from each population were used for both transcriptomic and metabolomic analyses to reduce inter-individual variation and enable direct integration of gene-expression and metabolite profiles. After dissection, the hepatopancreas tissue from each individual was divided into two aliquots, one for RNA extraction and the other for metabolite extraction. For shell microstructure and elemental composition analyses, shell samples were collected from a standardized region of each individual, approximately 5 mm above the operculum on the right side of the first whorl (*n* = 8). Four corresponding EDS regions were selected sequentially from the outer to the inner shell cross-section, representing the outer prismatic region, deeper prismatic or transitional region, crossed-lamellar region, and innermost shell region, respectively. The excised shell samples were washed with phosphate-buffered saline (PBS), immediately immersed in electron microscopy fixative, maintained at room temperature for 2 h, and then stored at 4 °C until further processing. A total of 6 snails were anesthetized using 200 mg/L MS-222, buffered to pH 7.0–7.5 before dissection. Hepatopancreas samples from 15 snails used for the determination of SOD, PK, and T-ATPase activities, as well as MDA content were stored at −20 °C. Hepatopancreas samples from 6 snails were promptly frozen in liquid nitrogen and preserved at −80 °C for transcriptome and metabolomic analyses.

### 2.3. Determination of Biochemical Parameter

Hepatopancreatic tissues were homogenized with ice-cold distilled water at a ratio of 1:9 (weight/volume). The supernatants were isolated using centrifugation (5000× *g*, 10 min, 4 °C) and thereafter kept at 4 °C. Protein concentrations in the supernatants were determined using the Bradford method [31]. The activities of SOD, PK, and T-ATPase, together with MDA content, were evaluated using commercial kits in accordance with the manufacturer’s instructions (Nanjing Jiancheng Bioengineering Institute, Nanjing, China).

### 2.4. Determination of Shell Microstructure and Elemental Composition

After primary fixation, the samples were rinsed three times with 0.1 M phosphate buffer (PB, pH 7.4) for 15 min each. After that, the samples were fixed in 1% osmium tetroxide made in 0.1 M phosphate buffer (PB, pH 7.4) for 1–2 h at room temperature in the dark. They were then washed three more times in 0.1 M phosphate buffer for 15 min each. Subsequently, the samples were dehydrated through a graded ethanol series (30%, 50%, 70%, 80%, 90%, 95%, 100%, and 100%), with each step lasting 15 min, followed by immersion in isoamyl acetate for 15 min. The dehydrated samples were dried using a critical point dryer, mounted on conductive carbon adhesive, and sputter-coated with gold for approximately 30 s to improve surface conductivity. Microstructural observations were performed using a Hitachi SU8100 scanning electron microscope (Su8100, Hitachi, Japan), and elemental composition was analyzed using an Oxford Ultim Max 100 energy-dispersive X-ray spectroscopy system (Oxford Instruments, Abingdon, UK).

Four corresponding EDS regions were selected sequentially from the outer to the inner shell surface: Spectrum 1, the outer mineralized region adjacent to the periostracum; Spectrum 2, the outer-middle shell region; Spectrum 3, the inner-middle shell region; and Spectrum 4, the innermost shell region adjacent to the inner surface. Corresponding regions at comparable relative positions were analyzed in both populations, and the elemental spectra and relative weight percentages were recorded. The detected elements mainly included C, nitrogen (N), O, sodium (Na), aluminum (Al), sulfur (S), chlorine (Cl), Ca, manganese (Mn), iron (Fe), and tellurium (Te).

### 2.5. Transcriptome Analysis

#### 2.5.1. RNA Extraction, Library Preparation, and Sequencing

Total RNA was extracted from hepatopancreas samples (*n* = 6 per group) using TRIzol reagent (Invitrogen, Carlsbad, CA, USA) according to the manufacturer’s instructions. To remove the residual genomic DNA, rDNase I (Ambion, Thermo Fisher Scientific, Waltham, MA, USA) was added to the purified RNA following the manufacturer’s guidelines. The quality and integrity of total RNA were assessed prior to library construction and sequencing, and RNA integrity number (RIN) values were recorded.

Transcriptomic libraries were constructed from individual RNA samples, and each biological sample was independently used for library preparation and sequencing without pooling. The libraries were sequenced on an Illumina NovaSeq X Plus platform (Illumina, San Diego, CA, USA) using paired-end 150 bp sequencing. Raw RNA-seq reads were filtered and trimmed using fastp v0.23.2 [32] with default settings. The clean reads were aligned to the reference genome in orientation mode using HISAT2 v2.2.1 (https://daehwankimlab.github.io/hisat2/, accessed on 20 June 2026). The reference genome of *C. cathayensis* was assembled in our laboratory and deposited in the Genome Sequence Archive (GSA) at the China National Center for Bioinformation (Project No. PRJCA066856) (https://ngdc.cncb.ac.cn/gsa/s/5C03xmg5, accessed on 20 June 2026). The mapped reads from each sample were assembled using StringTie v2.2.1 (https://ccb.jhu.edu/software/stringtie/index.shtml?t=example, accessed on 20 June 2026) through a reference-based methodology. All hepatopancreas transcriptome datasets produced in this study have been deposited in the Sequence Read Archive (SRA) database of the National Center for Biotechnology Information (NCBI) under accession number PRJNA1478776 (https://www.ncbi.nlm.nih.gov/bioproject/?term=PRJNA1478776, accessed on 20 June 2026).

#### 2.5.2. Analysis of the Differential Expression and Enrichment of Genes

Gene expression levels were measured in fragments per kilobase of exon per million mapped reads (FPKM) using RSEM v.1.3.3. Differential expression analysis was conducted using DESeq2 v.1.10.1 based on raw read counts. *p* values were adjusted using the Benjamini–Hochberg FDR correction. Genes with an adjusted *p* value < 0.05 and |log2 fold change| ≥ 1 were considered differentially expressed genes (DEGs). To identify genes showing the strongest transcriptional differences between populations, a more stringent threshold of adjusted *p* < 0.0001 and |log2 fold change| ≥ 3 was subsequently applied. Genes with a mean FPKM ≥ 1 in at least one population were retained. The top 20 DEGs (10 most significantly upregulated and 10 most significantly downregulated genes) were selected according to adjusted *p* values and visualized in a heatmap. GO and KEGG enrichment analyses of DEGs were conducted using Goatools v.0.6.5 and Kobas v.2.1.1, respectively. Significantly enriched GO terms and KEGG pathways were identified as having an adjusted *p* < 0.05.

#### 2.5.3. Quantitative Real-Time PCR Analysis

Ten DEGs were chosen for quantitative real-time PCR (RT-qPCR) validation to evaluate the accuracy of the RNA-seq results. The primers used for the selected DEGs are listed in Table S1, with *β*-*actin* serving as the reference gene for RT-qPCR normalization. *β*-*actin* was selected because it has been widely used as a reference gene in RT-qPCR analyses of molluscan species [33]. Total RNA was derived from hepatopancreas samples using TRIzol reagent (CWBIO, Beijing, China). cDNA was synthesized using the HiFiScript gDNA Removal cDNA Synthesis Kit (CWBIO, China) and used as the template for amplification. RT-qPCR was performed using SYBR^®^ Premix Ex Taq kits (Takara, Berkeley, CA, USA) and Quant Studio Design and Analysis Software v1.1 (Applied Biosystems, Foster City, CA, USA). The amplification program consisted of an initial denaturation step at 95 °C for 5 min, followed by 40 cycles of denaturation at 95 °C for 5 s and annealing/extension at 50 °C for 30 s. A melting curve analysis was performed after amplification to confirm the specificity of PCR products. Relative expression levels were calculated using the Ct (2^−ΔΔCT^) values [34]. The consistency between RT-qPCR and RNA-seq results was assessed by Pearson correlation analysis based on the log_2_ fold changes in the validated genes and was visualized using GraphPad Prism 8.0.

### 2.6. Metabolomics Analysis

#### 2.6.1. Metabolite Extraction and Profiling

A total of twelve hepatopancreas samples were used for metabolomic analysis. Frozen hepatopancreas samples stored at −80 °C were thawed on ice. Approximately 50 mg of each sample was weighed into a 1.5 mL EP tube for metabolite extraction. Each tube was supplemented with 400 μL of a cooled methanol/water combination (4:1, *v*/*v*). The samples were ground into a homogeneous paste using a tissue crusher set to 50 Hz and −20 °C. After homogenization, the samples were incubated at −20 °C for 30 min and then centrifuged at 13,000× *g* for 15 min at 4 °C. The clear supernatant was transferred into LC-MS injection vials and maintained at low temperature before analysis. All the chemicals used in the LC-MS analysis were of the highest purity and were purchased from Fisher Scientific in Hampton, NH, USA. Methanol, acetonitrile, formic acid, and isopropanol used for LC-MS analysis were of LC-MS grade and purchased from Fisher Scientific (Hampton, NH, USA). Chromatographic separation was performed using an ACQUITY UPLC HSS T3 (100 mm × 2.1 mm i.d., 1.8 μm; Waters, Milford, CT, USA). The mobile phases comprised solvent A, deionized water with 0.1% formic acid, and solvent B, a 1:1 (*v*/*v*) mixture of acetonitrile and isopropanol containing 0.1% formic acid. The column temperature was sustained at 40 °C, the injection volume was 10 μL, and the flow rate was established at 0.4 mL/min. The gradient elution program was as follows: 0–3 min, 95–80% A; 3–9 min, 80–55% A; 9–13 min, 55–5% A; 13–15 min, 5–95% A; and 15–16 min, 95% A. A scan range of 50–1200 *m*/*z* was used to set the MS conditions. The normalized collision energy was established at 20, 40 and 60 eV. In the positive mode (ESI+), the IonSpray floating voltage was +5500 V, whereas in negative mode (ESI−), it was −4500 V. A quality control (QC) sample was used during the analysis to make sure that the method would work the same way every time.

#### 2.6.2. Bioinformatics Analysis of Metabolomics Data

The Progenesis QI v 2.4 (Waters, Milford, MA, USA) was employed to preprocess the raw data from all LC-MS analysis. The following databases were utilized for metabolic pathway identification: HMDB (http://www.hmdb.ca/, accessed on 21 June 2026), METLIN (https://metlin.scripps.edu/, accessed on 21 June 2026), and KEGG (https://www.genome.jp/kegg/, accessed on 21 June 2026). Using ropls software (v. 1.6.2), we conducted studies involving many variables, including principal component analysis (PCA) and orthogonal partial least-squares discriminating analysis. A 200-permutation test was also used to validate the orthogonal partial least squares discriminant analysis (OPLS-DA) models. The OPLS-DA model’s VIP values (VIP > 1) and *p* values < 0.05 were used as screening criteria to discover possible biomarkers and differential metabolites. Using MetaboAnalyst [35] in conjunction with Fisher’s exact test for statistical evaluation, the contributions of the modified metabolites to KEGG pathways were examined afterwards. Metabolites with VIP > 1, *p* value < 0.05, and fold change (FC) ≥ 2 or FC ≤ 0.5 were classified as differentially enriched metabolites (DEMs).

### 2.7. Integrated Analysis of Transcriptome and Metabolome

Integrated transcriptomic and metabolomic analysis was conducted using the KEGG pathways that were significantly enriched in both datasets. The resulting correlation matrix was then visualized as a heatmap. The top 20 DEGs were selected based on adjusted *p* values, including the 10 most significantly upregulated and 10 most significantly downregulated genes, for visualization of expression patterns. The top 20 DEMs were selected based on VIP values, *p* values, and fold-change values. Pearson correlation analysis was performed between DEGs and DEMs using the expression levels of genes and relative abundance of metabolites from the same biological samples. For correlation analysis, the top 20 DEGs and DEMs were selected based on their statistical significance and contribution to population differentiation. Pearson correlation coefficients were then calculated between the selected genes and metabolites.

### 2.8. Data Analysis

Data normality and homogeneity of variance were assessed using the Shapiro-Wilk test and Levene’s test, respectively. Differences between the high-altitude and low-altitude populations were evaluated using an independent-samples *t*-test when the assumptions of normality and homogeneity of variance were met. Welch’s *t*-test was applied when the assumption of equal variances was violated. Statistical significance was set at *p* < 0.05, and the results were expressed as the mean ± standard deviation (SD).

## 3. Results

### 3.1. Differences in Water Temperature and Dissolved Oxygen Between the Two Sampling Sites

**Figure 1:**
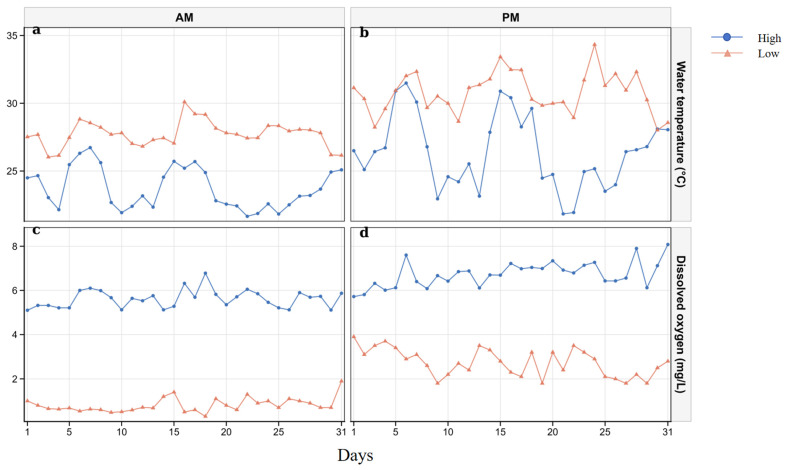
Daily variations in water temperature and dissolved oxygen at the high-and low-altitude sampling sites during July. Water temperature and dissolved oxygen were measured daily at two fixed time points (06:00 and 14:00) from 1 to 31 July 2025 at both sampling sites. AM, morning measurement (06:00); PM, afternoon measurement (14:00). High, high-altitude population; Low, low-altitude population. (**a**,**b**) Daily variations in water temperature at 06:00 (AM) and 14:00 (PM), respectively. (**c**,**d**) Daily variations in dissolved oxygen at 06:00 (AM) and 14:00 (PM), respectively.

Water temperature and dissolved oxygen exhibited distinct temporal patterns between the high- and low-altitude sampling sites during July 2025 (Figure 1). Throughout most of the monitoring period, water temperature was higher at the low-altitude site than at the high-altitude site during both the morning and afternoon periods. Morning water temperature at the high-altitude site generally ranged from approximately 22 to 27 °C, whereas that at the low-altitude site remained mostly between 26 and 30 °C. Afternoon water temperature showed greater day-to-day variability, particularly at the high-altitude site, where several pronounced increases and decreases were observed. In contrast, afternoon water temperature at the low-altitude site generally remained between approximately 29 and 34 °C.

Dissolved oxygen displayed an opposite spatial pattern. Morning dissolved oxygen concentrations at the high-altitude site were generally maintained at approximately 5–7 mg/L, whereas those at the low-altitude site remained mostly below 2 mg/L. Afternoon dissolved oxygen concentrations were higher than the corresponding morning values at both sites, reaching approximately 6–8 mg/L at the high-altitude site and 2–4 mg/L at the low-altitude site. Overall, the high-altitude site was characterized by lower water temperatures, greater short-term thermal fluctuations, and substantially higher dissolved oxygen concentrations than the low-altitude site.

### 3.2. Biochemical Parameter Assays

**Figure 2:**
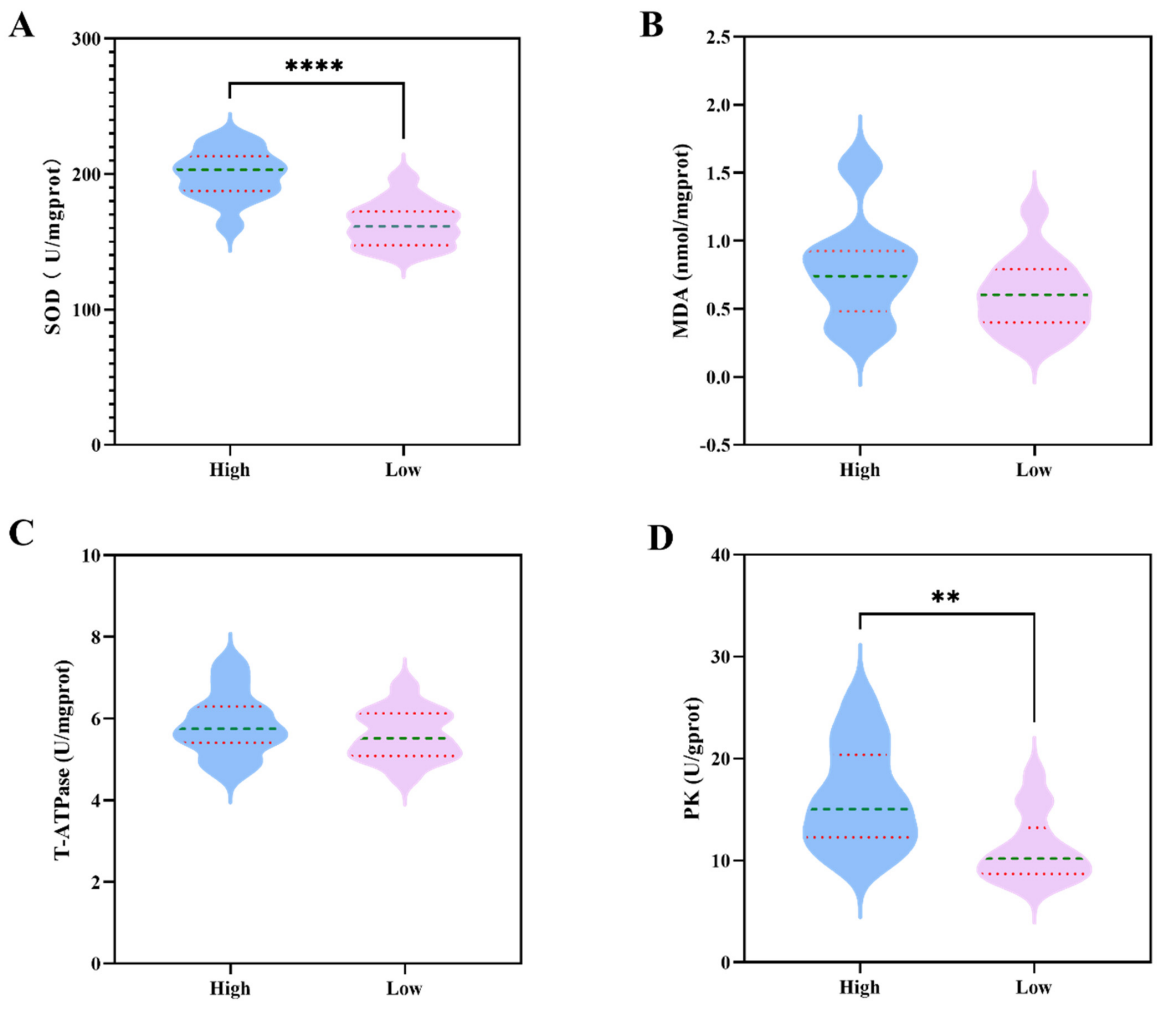
Biochemical parameters in the hepatopancreas of high-altitude and low-altitude populations of *C. cathayensis*. (**A**) superoxide dismutase (SOD); (**B**) malondialdehyde (MDA); (**C**) total ATPase (T-ATPase); (**D**) pyruvate kinase (PK). Values are expressed as mean ± SD, *n* = 15. ** *p* < 0.01, **** *p* < 0.0001. The green dashed line represents the median, and the red dotted lines represent the first and third quartiles. High, high-altitude population; Low, low-altitude population.

The SOD activity was significantly higher in the high-altitude population than in the low-altitude population (*p* < 0.0001). In contrast, the MDA content showed no significant difference between the two populations. Regarding energy metabolism, PK activity was significantly elevated in the high-altitude population compared with the low-altitude population (*p* < 0.01). However, T-ATPase activity exhibited no significant differences between the two populations (Figure 2).

### 3.3. Shell Microstructure and Elemental Composition

**Figure 3:**
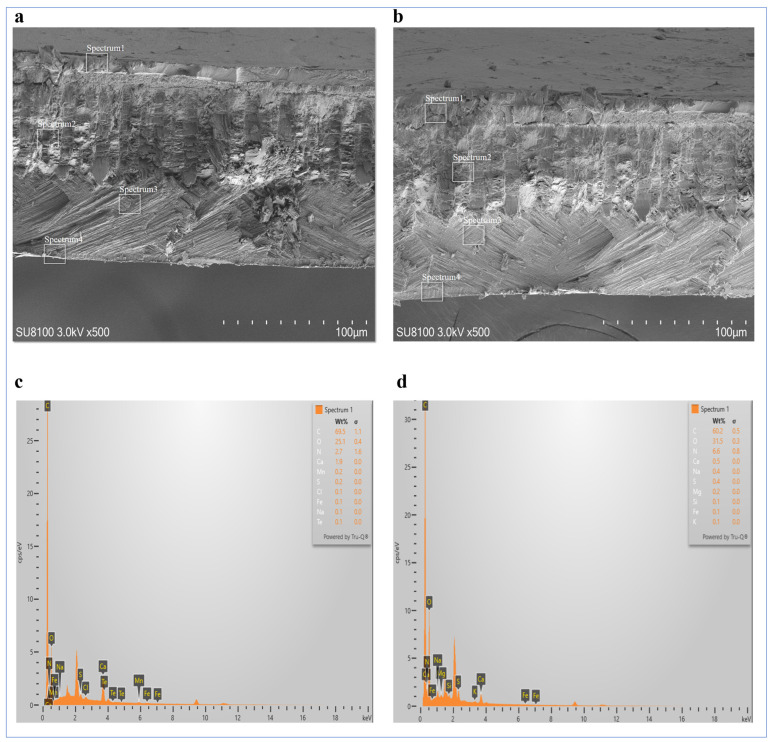
SEM images and EDS analyses of shell cross-sections from the high-altitude and low-altitude populations of *C. cathayensis*. (**a**) SEM image of the shell cross-section from the high-altitude population; (**b**) SEM image of the shell cross-section from the low-altitude population; (**c**) representative EDS spectrum of the high-altitude population; and (**d**) representative EDS spectrum of the low-altitude population. Four corresponding EDS regions were selected sequentially from the outer to the inner shell surface: Spectrum 1, the outer mineralized region adjacent to the periostracum; Spectrum 2, the outer-middle shell region; Spectrum 3, the inner-middle shell region; and Spectrum 4, the innermost shell region adjacent to the inner surface. Regions assigned the same spectrum number represented comparable positions and relative depths within the shell cross-sections of the two populations. Elemental contents are expressed as weight percentages (wt%).

The shell cross-sections of both the high-altitude and low-altitude populations of *C. cathayensis* showed a distinct layered structure under scanning electron microscopy (Figure 3). Four representative regions were selected for EDS analysis in each population. A total of 11 elements were detected in both populations. In the high-altitude population, the detected elements were C, N, O, Na, Al, S, Cl, Ca, Mn, Fe, and Te. In the low-altitude population, the detected elements were C, N, O, Na, Mg, Al, Si, S, K, Ca, and Fe. Among these elements, Ca, O, and C were the predominant components in both populations. The Ca content was higher in the high-altitude population (36.76 wt%) than in the low-altitude population (18.99 wt%), whereas the relative C content was lower in the high-altitude population (27.08 wt%) than in the low-altitude population (41.02 wt%). The O content was comparable between the two populations, with mean values of 32.49 wt% in the high-altitude population and 34.90 wt% in the low-altitude population.

### 3.4. Transcriptomics Analysis

**Figure 4:**
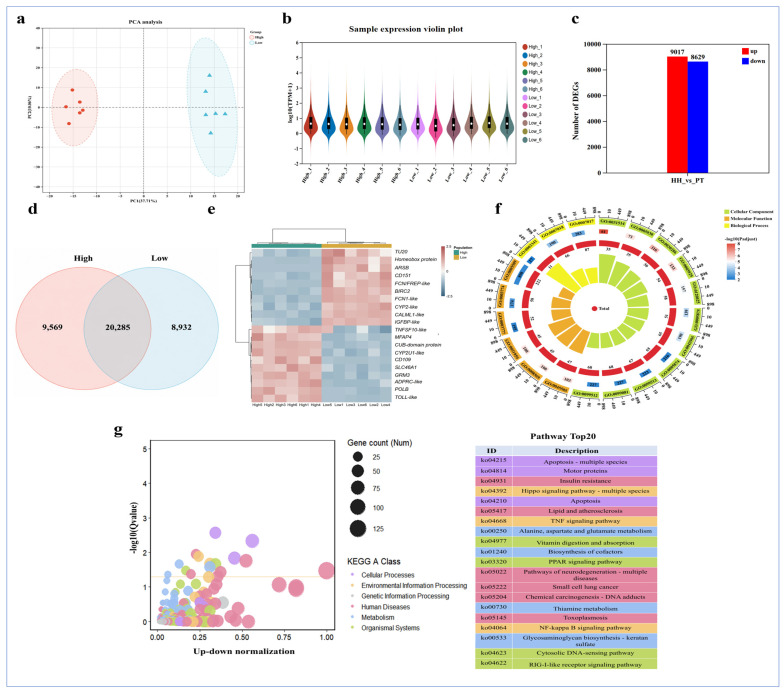
Transcriptome analysis of DEGs in the hepatopancreas of *C. cathayensis* between the high-altitude and low-altitude populations. (**a**) Principal component analysis showing the separation of transcriptomic samples between the high-altitude and low-altitude populations. (**b**) Violin plots of the different gene expressions; (**c**) Statistical summary of significant DEGs between the high-altitude and low-altitude populations; (**d**) Venn diagram showing the overlap of DEGs between the high-altitude and low-altitude populations. (**e**) Heatmap of the top 20 DEGs; (**f**) GO enrichment circle plot for the high-altitude versus low-altitude comparison; the first circle is the top-20 GO term of enrichment, and the outerside is the fit scale for the number of DEGs. Different colors represent different ontologies; the second circle is the number of the GO term in the background of the DEGs and the Q value. The longer the number, the longer the bar, and the smaller the Q value, the redder the color; the third circle is the proportion of upregulated and downregulated DEGs, the fourth circle is the rich factor value of each GO term; (**g**) Sankey diagram and KEGG enrichment analysis of representative differentially expressed genes between the high and low populations. The left panel shows representative differentially expressed genes, which are linked to the enriched KEGG pathways in the middle panel. The right panel shows the enrichment statistics of these pathways. The *x*-axis represents the rich factor, bubble size indicates the number of enriched genes, and bubble color represents the enrichment significance as −log10 (*p* value).

RNA-seq generated 531,434,204 raw reads, with GC content ranging from 41.32 to 42.66%. The average Q30 value exceeded 96.66%, and uniquely mapped reads accounted for more than 88.12%. The RNA samples used for library construction showed high integrity, with RNA integrity number (RIN) values ranging from 9.00 to 10.00 (Table S2). Principal component analysis (PCA) showed clear separation between the high-altitude and low-altitude populations, whereas biological replicates within each population clustered closely together, indicating relatively low inter-individual variation among transcriptomic samples (Figure 4a). A total of 86,693 genes were quantified using FPKM. A total of 3767 DEGs were identified between the high-altitude and low-altitude populations, with 2087 genes upregulated and 1680 genes downregulated in the high-altitude population compared with the low-altitude population (Figure 4c, Table S3). The expression patterns of the top 20 DEGs were visualized using a heatmap, showing distinct expression clusters between the high-altitude and low-altitude populations (Figure 4e). Several representative genes involved in intracellular transport, cellular regulation, and stress-associated responses showed marked expression differences between the two populations. Functional enrichment analysis based on GO and KEGG databases revealed that the DEGs identified in the high-altitude population compared with the low-altitude population were mainly enriched in calcium ion binding (GO:0005509), microtubule-based process (GO:0007017), dynein complex (GO:0030286), and cell projection (GO:0042995) (Figure 4f). KEGG enrichment analysis further showed that multiple environment-responsive pathways were significantly enriched, including the FoxO signaling pathway, apoptosis, motor proteins, alanine, aspartate and glutamate metabolism, and steroid hormone biosynthesis (Figure 4g).

### 3.5. Metabolome Analysis

**Figure 5:**
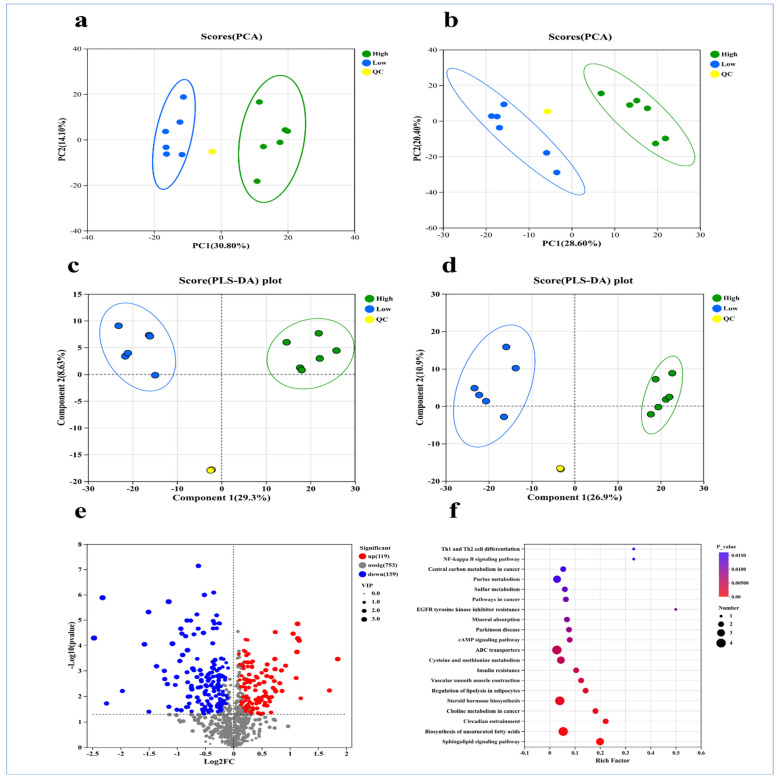
Metabolomic analysis of DEMs in the hepatopancreas of high-altitude and low-altitude populations of *C. cathayensis.* (**a**,**b**) PCA score of hepatopancreas metabolites ((**a**): positive ions, (**b**): negative ions). (**c**,**d**) OPLS-DA score of hepatopancreas metabolites ((**c**): positive ions, (**d**): negative ions). (**e**) Volcano plot of differential metabolites between the high-altitude and low-altitude populations; (**f**) Enrichment analysis of the KEGG pathway of DEMs (the top 20 most enriched term).

PCA was performed to assess the overall distribution and stability of the metabolomic data. All samples fell within the 95% Hotelling’s T-squared confidence ellipse, indicating good analytical stability. The PCA models showed R^2^X values of 0.563 and 0.585 in positive and negative ion modes, respectively (Figure 5a,b). To further characterize metabolic differences between the high-altitude and low-altitude populations, OPLS-DA was conducted after data normalization. In the OPLS-DA models, R^2^Y indicates the goodness of fit, whereas Q^2^ represents the predictive ability of the model. The OPLS-DA score plots showed clear separation between the two populations in both ionization modes, with model parameters of R^2^X = 0.991 and Q^2^ = 0.911 in ESI+ mode and R^2^X = 0.985 and Q^2^ = 0.915 in ESI− mode (Figure 5c,d). DEMs were selected based on variable importance in projection (VIP) values > 1 from the OPLS-DA model and Student’s *t*-test (*p* < 0.05). A total of 278 DEMs were identified between the high-altitude and low-altitude populations, including 119 upregulated and 159 downregulated metabolites in the high-altitude population relative to the low-altitude population (Figure 5e). Representative DEMs included L-serine, L-methionine, adenosine, sulfate, tetrahydrocorticosterone, dehydroepiandrosterone sulfate, dihomo-α-linolenic acid, 8-hydroxyadenine, dihydroxyacetone, PC (22:5/0:0), melatonin, secoisolariciresinol, glyceryl palmitoleate, and 10-hydroxydecanoic acid. The compounds were subsequently annotated using the Human Metabolome Database (HMDB). KEGG pathway enrichment analysis was performed using MetaboAnalyst 5.0 to ascertain metabolic pathways possibly influenced by altitude-related population variations. The DEMs were mainly enriched in purine metabolism, cysteine and methionine metabolism, glycerophospholipid metabolism, ABC transporters, steroid hormone biosynthesis, the sphingolipid signaling pathway, circadian entrainment, and biosynthesis of unsaturated fatty acids (Figure 5f and Table 1).

### 3.6. Verification of RNA-Seq by RT-qPCR

**Figure 6:**
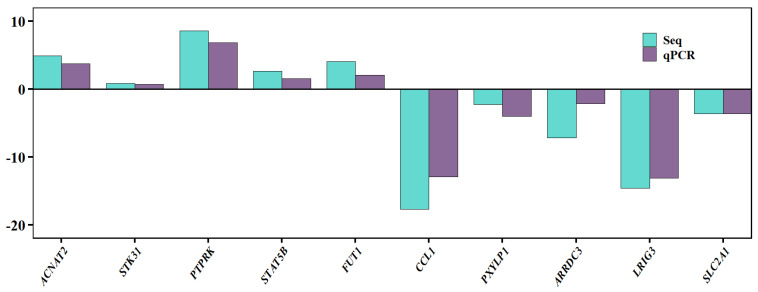
qPCR validation of RNA-Seq, including summary information of 10 selected DEGs.

**Figure 7:**
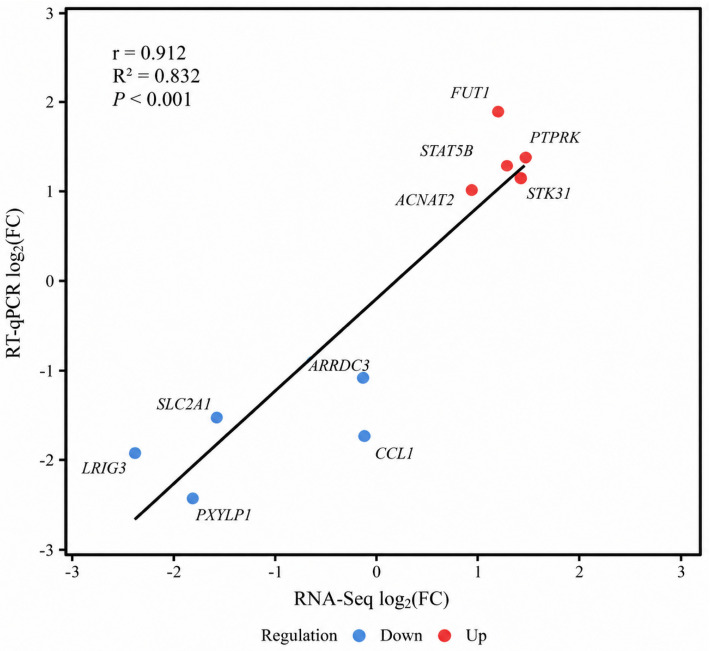
Correlation between RNA-seq and RT-qPCR expression changes in validated DEGs.

The RT-qPCR results were compared with the expression profiles obtained from RNA-seq analysis. Ten selected DEGs, including five upregulated genes (*FUT1*, *STAT5B*, *PTPRK*, *STK31*, and *ACNAT2*) and five downregulated genes (*SLC2A1*, *LRIG3*, *PXYLP1*, *ARDC3*, and *CCL1*) in the high-altitude population compared with the low-altitude population, showed consistent expression patterns between RT-qPCR and RNA-seq analyses (Figure 6). Furthermore, the fold changes obtained from RT-qPCR analysis showed a strong positive correlation with those derived from RNA-seq analysis (Pearson’s correlation coefficient, r = 0.912; R^2^ = 0.832; *p* < 0.001), indicating a high degree of consistency between the two approaches (Figure 7).

### 3.7. Integrated Analysis of Transcriptomics and Metabolomics

**Figure 8:**
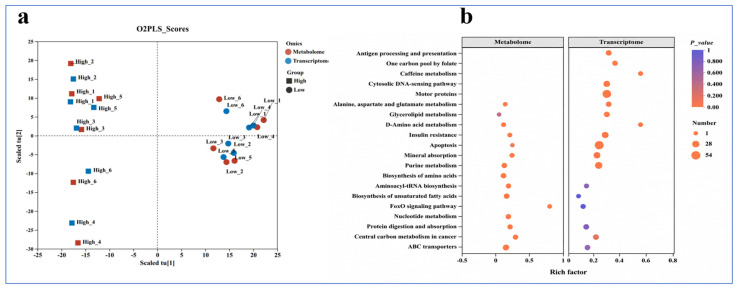
Integrated transcriptomic and metabolomic analysis of the high-altitude and low-altitude populations of *C. cathayensis*. (**a**) O2PLS score plot showing the relationship between the transcriptomic and metabolomic datasets. Colors represent omics types, with red indicating the metabolome and blue indicating the transcriptome. Shapes represent population groups, with squares indicating the high-altitude population and circles indicating the low-altitude population; (**b**) KEGG pathway enrichment analysis of DEMs and DEGs. The *x*-axis represents the rich factor, bubble size indicates the number of enriched metabolites or genes, and bubble color represents the *p* value.

To further elucidate the molecular regulatory mechanisms underlying altitude-related adaptation in *C. cathayensis*, integrated transcriptomic and metabolomic analyses were performed between the high-altitude and low-altitude populations. The O2PLS analysis demonstrated strong association between the transcriptome and metabolomic datasets in the high-altitude and low-altitude populations of *C. cathayensis* (Figure 8a). Furthermore, Pearson correlation analysis was performed to evaluate the relationships between DEGs and DEMs, and a strong correlation was observed between the top 20 DEGs and DEMs in the high-altitude and low-altitude populations of *C. cathayensis*. The Pearson correlation coefficients and corresponding significance values are provided in Supplementary Table S4. Integrated pathway analysis identified four common pathways in the transcriptomic and metabolomic datasets, including purine metabolism, D-amino acid metabolism, insulin resistance, and alanine, aspartate and glutamate metabolism (Figure 8b). Among these pathways, nucleotide metabolism, amino acid metabolism, and metabolic regulation were prominently represented.

## 4. Discussion

In the present study, we demonstrated that high-altitude and low-altitude populations of *C. cathayensis* exhibited distinct physiological and molecular responses under contrasting environmental conditions. The high-altitude population showed enhanced antioxidant capacity and energy metabolism, accompanied by differences in shell biomineralization characteristics between the two populations. Integrated transcriptomic and metabolomic analyses further revealed coordinated regulation of stress-responsive processes and metabolic pathways associated with altitude-related environmental variation. Our findings provide new insights into altitude-associated physiological and molecular responses in freshwater snails and broaden the current understanding of environmental responses in mollusks.

### 4.1. Environmental Temperature and Dissolved Oxygen Differences Between Altitudinal Habitats

Temperature and dissolved oxygen (DO) are critical environmental factors influencing physiological performance, metabolic regulation, and habitat suitability in freshwater mollusks. In the present study, the high-altitude and low-altitude populations of *C. cathayensis* inhabited contrasting paddy-field environments. The high-altitude habitat exhibited lower overall water temperatures but greater short-term thermal fluctuations, while maintaining higher DO concentrations compared with the low-altitude habitat. In contrast, the low-altitude habitat showed warmer water conditions accompanied by reduced DO availability. Previous studies in paddy-field ecosystems have demonstrated that DO dynamics are regulated by the interaction between physical conditions and biological processes. In flooded paddy fields, DO concentrations fluctuate temporally due to the balance between photosynthetic oxygen production and oxygen consumption associated with respiration and microbial activity. Daytime photosynthetic activity can increase DO availability, whereas respiration and microbial oxygen consumption may reduce oxygen levels during periods of reduced light or enhanced biological activity [36,37]. Therefore, oxygen conditions in paddy habitats are determined by both regional climatic factors and local ecosystem processes.

Water temperature represents an important factor regulating DO dynamics in shallow paddy ecosystems. Higher temperatures can reduce oxygen retention capacity and increase biological oxygen demand, thereby contributing to lower DO availability [36]. Accordingly, the warmer conditions observed in the low-altitude paddy habitat may have promoted higher oxygen consumption and resulted in the lower DO concentrations detected in this population. In contrast, the lower temperatures observed in the high-altitude habitat may have enhanced oxygen retention and reduced metabolic oxygen consumption, contributing to the relatively higher DO concentrations observed in this site. However, oxygen availability in high-altitude aquatic environments is highly dependent on elevation and local habitat characteristics. Extreme high-altitude ecosystems, such as Tibetan Plateau lakes and alpine streams (>4000 m), often experience oxygen limitation due to reduced atmospheric oxygen pressure despite low temperatures [38,39]. In contrast, the present study was conducted in a moderate-elevation paddy habitat (~1000 m), where atmospheric oxygen limitation is relatively weak and local environmental processes may play a more important role in regulating DO dynamics.

### 4.2. Antioxidant Defense and Metabolic Regulation Under Environmental Stress

SOD and PK activities were measured to evaluate antioxidant defense and glycolytic metabolism, respectively, whereas T-ATPase activity was used as an indicator of ATP hydrolysis and ATP-dependent ion transport. As a commonly used indicator of lipid peroxidation and oxidative damage, MDA reflects the extent of oxidative injury to cellular membranes [40,41]. In the present study, SOD and PK activities in the hepatopancreas were significantly higher in the high-altitude population than in the low-altitude population, whereas MDA content and T-ATPase activity did not differ significantly between the two populations. In *C. cathayensis*, previous studies have demonstrated that environmental stress induces changes in antioxidant enzyme activities and oxidative damage indicators, with SOD serving as an important component of antioxidant defense and MDA reflecting the extent of lipid peroxidation and cellular oxidative injury [19]. Therefore, the markedly higher SOD activity observed in the hepatopancreas of the high-altitude population (*p* < 0.0001) suggests that *C. cathayensis* inhabiting high-altitude environments possesses stronger enzymatic antioxidant defenses. Notably, the unchanged MDA content indicates that the high-altitude population did not experience greater lipid peroxidation in the hepatopancreas despite its elevated SOD activity. The higher SOD activity, together with the unchanged MDA content, suggests that the high-altitude population may have an enhanced capacity for ROS removal without a concomitant increase in lipid peroxidation. Overall, the combination of increased SOD activity and stable MDA content suggests that the high-altitude population of *C. cathayensis* may maintain redox homeostasis through enhanced antioxidant activity.

PK catalyzes the final glycolytic reaction, in which phosphoenolpyruvate and ADP are converted into pyruvate and ATP [42]. In the present study, the marked increase in PK activity indicates that carbohydrate metabolism was adjusted in the high-altitude population. Under reduced oxygen availability, enhanced PK activity is generally associated with increased glycolytic ATP production and compensatory energy metabolism [43]. In largemouth bass (*Micropterus salmoides*), hypoxia enhanced the glycolysis/gluconeogenesis pathway and increased the expression and activity of essential glycolytic enzymes, including HK, PFK, and PK in the gills [43]. In the oyster *Crassostrea hongkongensis*, PK activity showed a time-dependent increase under prolonged hypoxic exposure, indicating metabolic reorganization during sustained oxygen limitation [44]. Although direct evidence from high- and low-altitude snail populations remains limited, these findings support the interpretation that the higher PK activity observed in the hepatopancreas of the high-altitude population of *C. cathayensis* may reflect enhanced glycolytic capacity in response to altitude-associated energetic demands. Together with the elevated SOD activity, this result suggests that antioxidant defense and glycolytic metabolism are both involved in the physiological differentiation of the high-altitude population. By contrast, T-ATPase activity showed no significant difference between the high-altitude and low-altitude populations. T-ATPase reflects the overall ATP-hydrolyzing capacity of ATPase-related enzymes, and the unchanged activity indicates that ATPase-associated ATP hydrolysis in the hepatopancreas remained relatively stable between the two populations. ATPase activity is involved in ion transport and cellular energy expenditure [40,41,45], but its response to environmental stress can vary with species, tissue type, stressor, and the ATPase endpoint examined [41,45,46,47]. For example, T-ATPase activity increased transiently in the gills of Yesso scallop (*Patinopecten yessoensis*) under acute high-temperature stress, indicating that ATPase-associated ATP hydrolysis in mollusks can respond to environmental stress in a tissue-dependent manner [41]. In rainbow trout (*Oncorhynchus mykiss*) hepatocytes, acute hypoxia transiently reduced Na^+^/K^+^-ATPase transport activity without a parallel change in its hydrolytic function, suggesting that ATPase-related responses may differ according to the functional endpoint examined [46]. In the land snail *Otala lactea*, Na^+^/K^+^-ATPase activity was also regulated differently between the hepatopancreas and foot muscle during estivation [45]. Therefore, the enzymatic differences between the high-altitude and low-altitude populations of *C. cathayensis* were mainly reflected in antioxidant defense and glycolytic metabolism, rather than in T-ATPase-related ATP hydrolysis. These results suggest that altitude-associated physiological differentiation in *C. cathayensis* may involve selective regulation of specific enzymatic pathways.

### 4.3. Shell Biomineralization Responses Under Contrasting Altitude Environments

Molluscan shells are biomineralized formations mostly consisting of calcium carbonate (CaCO_3_), together with a small proportion of organic matrix that participates in CaCO_3_ deposition and shell microstructural organization [24]. Previous studies have shown that the shell of *Cipangopaludina chinensis* is mainly composed of calcium carbonate, exhibiting a cross-lamellar architecture covered by a periostracum, with aragonite identified as the dominant mineral phase [23]. In the present study, the high-altitude and low-altitude populations of *C. cathayensis* originated from contrasting altitude-associated habitats, and their shells differed in thickness, microstructure, and elemental composition. SEM observations showed that both populations exhibited a distinct layered architecture in shell cross-sections, but the high-altitude population had a thinner shell and showed differences in shell-layer organization compared with the low-altitude population. EDS analysis detected 11 elements in each population, with Ca, O, and C being the dominant elements in both groups. The mean Ca content was higher in the high-altitude population than in the low-altitude population, whereas the mean C content was lower in the high-altitude population. The O content was relatively comparable between the two populations. These results indicate that the two populations differed not only in shell thickness and microstructure but also in the relative elemental composition of the shell cross-sections. Shell properties are not determined by thickness alone, but are also influenced by mineral composition, crystal arrangement, porosity, and microstructural organization [27,48,49,50]. Environmental calcium and habitat physicochemical conditions can further modify shell formation in freshwater gastropods. In *Biomphalaria sudanica*, calcium concentration affected shell size, inorganic dry weight, and crushing resistance [51]. Calcium availability has also been shown to modify predator-induced shell defenses in freshwater snails [52]. In addition, studies of freshwater gastropods have also indicated that shell microstructure can vary with environmental conditions, particularly temperature. Studies on freshwater gastropods have demonstrated that thermal conditions can influence shell formation processes and microstructure. In *Viviparus viviparus*, temperature variation affected shell growth and crossed-lamellar microstructural organization, indicating that thermal environments can leave measurable effects on shell biomineralization [53]. Therefore, the thinner shell and altered shell-layer organization observed in the high-altitude population of *C. cathayensis* may partly reflect long-term responses to altitude-associated environmental conditions, including differences in temperature and mineral availability. However, shell biomineralization is a complex process involving interactions between inorganic mineral deposition and organic matrix formation. Because carbon detected by EDS may originate from both inorganic carbonate components and organic shell matrices, the higher Ca/C ratio observed in the high-altitude population does not necessarily indicate enhanced calcium carbonate deposition. It may reflect differences in the relative proportions of mineral components and organic matrix within the shell. Overall, the observed differences in shell thickness, microstructure, and elemental composition suggest that high-altitude and low-altitude populations of *C. cathayensis* may employ distinct shell biomineralization strategies in response to contrasting environmental conditions.

### 4.4. Transcriptomic Regulation of Intracellular Transport and Stress Responses Under Altitude-Associated Stress

**Figure 9:**
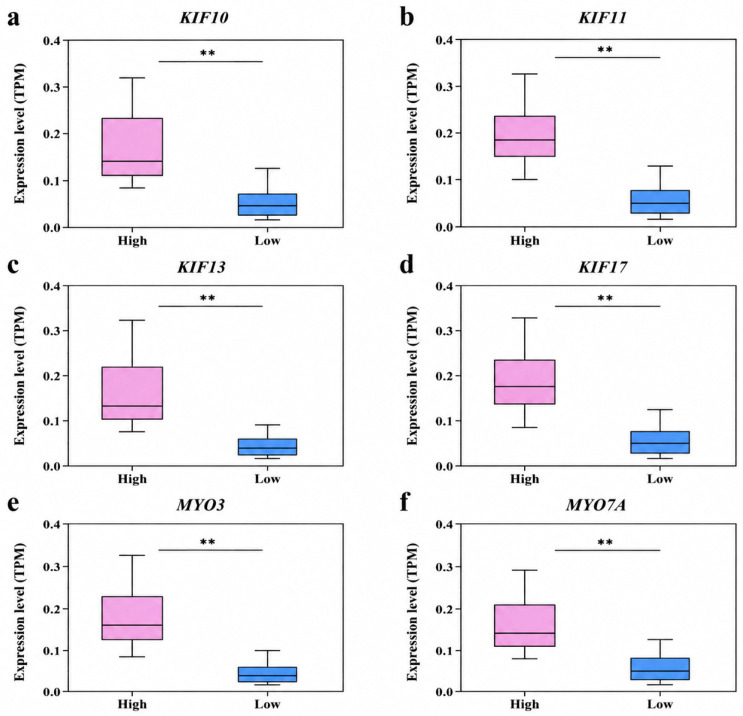
Expression levels of motor protein-related genes in the high-altitude and low-altitude populations of *C. cathayensis*. The expression levels of *KIF10* (**a**), *KIF11* (**b**), *KIF13* (**c**), *KIF17* (**d**), *MYO3* (**e**) and *MYO7A* (**f**) are shown as TPM values. Asterisks indicate significant differences between the two populations: * *p* < 0.05; ** *p* < 0.01; *** *p* < 0.001. High, high-altitude population; Low, low-altitude population.

**Figure 10:**
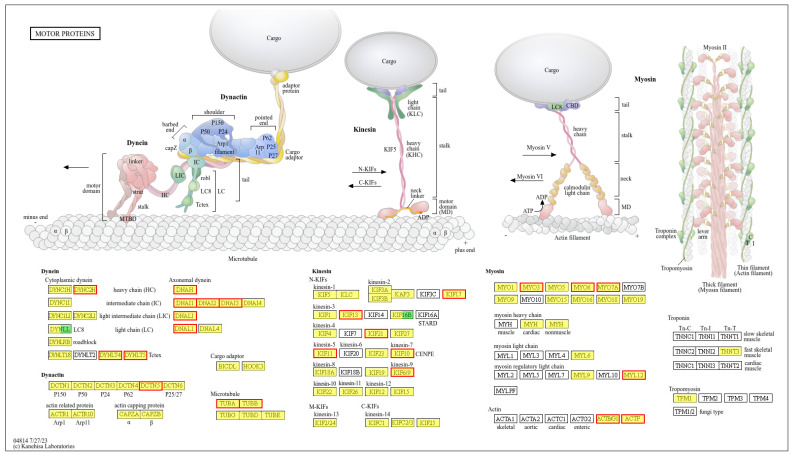
KEGG pathway map of motor proteins showing differentially expressed genes between the high-altitude and low-altitude populations of *C. cathayensis.* Yellow boxes indicate genes involved in the motor protein pathway. Red boxes indicate genes with upregulated expression, and green boxes indicate genes with downregulated expression. Arrows indicate the direction of motor protein movement along cytoskeletal filaments.

To investigate the molecular mechanisms associated with altitude-related differentiation in *C. cathayensis*, transcriptome profiling was performed in the hepatopancreas. A total of 3767 differentially expressed genes (DEGs) were identified, indicating extensive transcriptional remodeling associated with intracellular transport, stress responses, apoptosis regulation, and cellular maintenance. These results suggest that long-term exposure to altitude-associated environmental conditions induces broad molecular adjustments to maintain cellular homeostasis. KEGG enrichment analysis showed that DEGs were prominently enriched in the motor proteins pathway. Motor proteins are ATP-dependent molecular machines that convert chemical energy from ATP hydrolysis into mechanical force and mediate intracellular cargo transport, organelle positioning, and cytoskeletal organization [54]. Kinesins and myosins are major classes of cytoskeleton-associated motor proteins, with kinesins primarily responsible for microtubule-dependent transport of organelles, protein complexes, and mRNAs, whereas myosins regulate actin-based intracellular movement and cellular remodeling [55,56]. In the present study, several representative kinesin family genes, including *KIF10*, *KIF11*, *KIF13*, and *KIF17*, as well as myosin family genes such as *MYO3* and *MYO7A*, were more highly expressed in the high-altitude population than in the low-altitude population (Figure 9). These transcriptional changes suggest enhanced regulation of cytoskeleton-dependent intracellular transport under altitude-associated stress. Similar cytoskeleton-associated responses have also been reported in mollusks exposed to environmental stress. For example, transcriptomic analyses of pearl oyster (*Pinctada fucata martensii*) under long-term hypoxia revealed significant enrichment of microtubule-based processes, kinesin complexes, and ATPase activity, suggesting that cytoskeletal remodeling and intracellular transport regulation are important components of molluscan stress responses [57]. In addition, hypoxia exposure induced significant alterations in myosin complex and actin cytoskeleton-related processes in pearl oysters, further supporting the involvement of motor proteins in environmental stress responses [58]. Therefore, altered expression of motor protein genes, particularly *KIF11* and *KIF13*, may facilitate intracellular organization and functional maintenance in the high-altitude population of *C. cathayensis* (Figure 10).

In addition to intracellular transport, genes involved in apoptosis regulation and cellular stress responses also showed differential expression between populations. Caspases are key regulators of programmed cell death, with *CASP2* involved in apoptotic initiation and *CASP3* acting as an executioner caspase during apoptosis [59,60]. However, *BCL*-*2*, an important anti-apoptotic regulator involved in maintaining mitochondrial membrane integrity, was also downregulated in the high-altitude population. This contrasting expression pattern suggests that apoptotic regulation may involve a complex balance between apoptotic sensitivity and execution. Specifically, reduced *BCL*-*2* expression may increase cellular susceptibility to apoptotic stimuli, whereas decreased *CASP2* and *CASP3* expression may limit downstream caspase-mediated apoptotic execution. Therefore, the observed changes in apoptosis-related genes likely represent a fine-tuned regulation of cellular homeostasis rather than a simple activation or inhibition of apoptosis under altitude-associated environmental conditions [61]. Previous studies have shown that hypoxia under high-altitude conditions can affect apoptosis-related pathways. In mice, high-altitude cerebral hypoxia promoted mitochondrial dysfunction and neuronal apoptosis, and chronic hypobaric hypoxia increased the number of TUNEL-positive neurons, enhanced caspase-3 activation, and shifted the balance between pro- and anti-apoptotic *BCL*-*2* family members [62,63]. In the present study, the lower expression of *CASP2* and *CASP3* in the high-altitude population of *C. cathayensis* may reflect reduced transcriptional activation of the caspase cascade under long-term altitude-associated habitat conditions. In addition, *GADD45A* was differentially expressed between the two populations. *GADD45A* is a stress-responsive gene involved in DNA damage response, cell-cycle control, and cellular stress signaling [64]. In the Andean killifish *Orestias ascotanensis*, genes associated with DNA repair and genome stability were reported to be under positive selection, suggesting that cellular maintenance systems may contribute to adaptation to high-altitude environments [65]. The differential expression of *GADD45A* in *C. cathayensis* may therefore reflect adjustment of genome-protective mechanisms in response to long-term altitude-associated habitat conditions.

### 4.5. Metabolic Remodeling of Redox, Nucleotide, and Membrane Homeostasis Under Altitude-Associated Stress

Metabolomic profiling further revealed altitude-associated metabolic differences between the high-altitude and low-altitude populations of *C. cathayensis*. A total of 278 differentially expressed metabolites (DEMs) were identified across the two ionization modes, including 119 upregulated and 159 downregulated metabolites. These metabolites were mainly enriched in pathways related to amino acid metabolism, steroid hormone biosynthesis, ABC transporters, circadian entrainment, and unsaturated fatty acid biosynthesis, indicating broad metabolic remodeling associated with altitude-associated environmental conditions. In the high-altitude population, L-serine, sulfate, and L-methionine were increased, showing changes in amino acid and sulfur-associated metabolism. L-serine is a non-essential amino acid derived mainly from glycolytic intermediates and serves as an important carbon donor for one-carbon metabolism. One-carbon metabolism provides one-carbon units for nucleotide synthesis and contributes to mitochondrial redox balance under hypoxic conditions [66]. A comparative metabolomic study of the high-altitude lizard (*Phrynocephalus vlangalii*) also reported altitude-related differences in amino acid- and lipid-related metabolites, including an increase in L-serine [67]. In the present study, the elevated L-serine level in the high-altitude population was associated with nucleotide turnover and redox maintenance in the hepatopancreas. Because high-altitude environments are typically characterized by chronic hypoxia and oxidative challenge, the elevation of serine- and methionine-related metabolites may reflect a compensatory strategy to support antioxidant defense and cellular maintenance [68]. Another notable feature was the change in nucleoside- and vitamin-related metabolites, especially the increase in adenosine and riboflavin. Adenosine is a well-established hypoxia-responsive metabolite and plays a central role in oxygen sensing, vasoregulation, anti-inflammatory signaling, and metabolic protection. Studies in humans have shown that plasma adenosine rises rapidly upon high-altitude exposure and contributes to acclimatization to hypoxia. Therefore, the upregulation of adenosine in the high-altitude population in the present study likely reflects an adaptive response to long-term oxygen limitation. The elevation of riboflavin also appears noteworthy. Riboflavin is the precursor of FMN and FAD, two flavin cofactors required for mitochondrial electron transfer, oxidative metabolism, and a wide range of cellular redox reactions [69].

The fatty acid profiles differed between the high-altitude and low-altitude populations. Several unsaturated fatty acids, including 11,14-eicosadienoic acid and dihomo-α-linolenic acid, were decreased in the high-altitude population, whereas 13,16-docosadienoic acid was increased. These changes showed that the two populations differed in unsaturated fatty acid composition. Recent work on fish also showed that cold stress is accompanied by lipid metabolic adjustment, especially changes in phospholipid and unsaturated fatty acid composition [70]. In mollusks, low-temperature exposure altered lipid and fatty acid profiles in the noble scallop (*Chlamys nobilis*), and polyunsaturated fatty acids were considered important for resistance to low-temperature stress [71]. Therefore, the altered abundance of unsaturated fatty acids in *C. cathayensis* was associated with membrane-level metabolic adjustment under altitude-related temperature and oxygen conditions.

**Figure 11:**
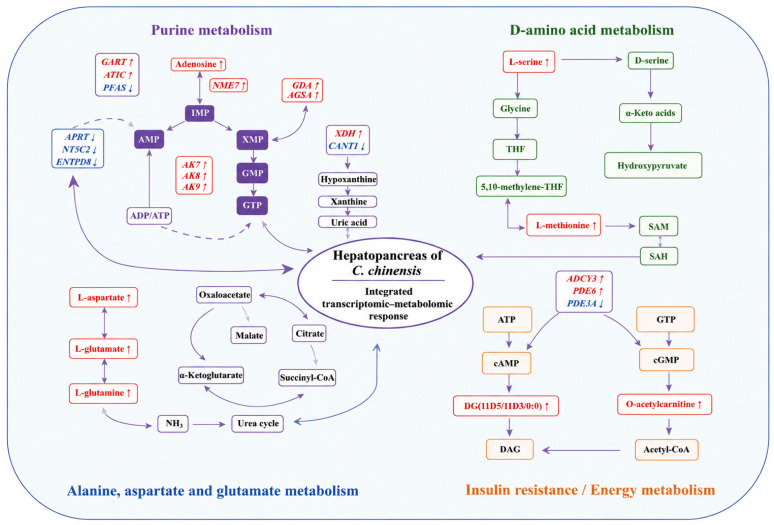
Integrated pathway network of DEGs and DAMs based on combined transcriptomic and metabolomic analyses in *C. cathayensis*. The Red and blue indicate up-regulated and down-regulated genes or metabolites, respectively. Green and orange boxes represent D-amino acid metabolism and insulin resistance/energy metabolism modules, respectively. Red arrows indicate up-regulated components, whereas blue arrows indicate down-regulated components. The direction of the arrows represents the metabolic conversion or regulatory relationship among the components within each pathway.

The integrated transcriptome and metabolome analysis revealed that purine metabolism, D-amino acid metabolism, and alanine, aspartate and glutamate metabolism were commonly enriched pathways associated with population differences in *C. cathayensis*. These pathways were mainly associated with nucleotide turnover, amino acid metabolism, lipid-associated energy regulation, and intracellular signaling in the hepatopancreas. In the high-altitude population, adenosine was increased, together with differential expression of several purine-associated genes, including upregulated *GART*, *ATIC*, *AGSA*, *GDA*, *AK7*, *AK8*, *AK9*, *NME7*, *NTPCR*, and several *XDH loci*, as well as downregulated *PFAS*, *APRT*, *NT5C2*, *ENTPD8*, and *CANT1*. These gene–metabolite changes showed that purine metabolism differed between the two populations. Purine metabolism is closely linked to nucleotide turnover, ATP metabolism, and cellular responses to reduced oxygen availability. Studies on high-altitude aquatic animals have also shown that plateau adaptation involves changes in energy metabolism, transport processes, antioxidant defense, and cellular maintenance. For example, transcriptomic analysis of the Tibetan Schizothoracinae fish (*Gymnocypris przewalskii*) revealed changes related to energy metabolism and transport during adaptation to high-altitude aquatic environments [72]. In another plateau fish, *Gymnocypris eckloni*, hypoxia exposure induced a shift from aerobic oxidation toward anaerobic glycolysis, reduced major energy-consuming processes, and enhanced antioxidant defense [73]. Therefore, the increased adenosine level and differential expression of purine-metabolic genes in *C. cathayensis* were associated with altered nucleotide turnover, energy demand, and redox balance in the high-altitude population. The enrichment of D-amino acid metabolism and alanine, aspartate and glutamate metabolism further showed that amino acid metabolism differed between the two populations. The high-altitude population had higher levels of L-serine and L-methionine. L-serine provides one-carbon units for nucleotide synthesis and contributes to mitochondrial redox balance under hypoxic conditions [66], whereas L-methionine is involved in sulfur amino acid metabolism and antioxidant-related regulation [74]. One-carbon metabolism supplies carbon units for purine biosynthesis; therefore, the concurrent enrichment of purine metabolism and serine/methionine-associated pathways linked amino acid utilization with nucleotide metabolism in the high-altitude population. Changes in L-aspartate, L-glutamate, and L-glutamine may also reflect differences in carbon–nitrogen exchange and biosynthetic support in the hepatopancreas (Figure 11).

Overall, the integrated transcriptomic and metabolomic analysis showed that the high-altitude and low-altitude populations of *C. cathayensis* differed mainly in purine metabolism, amino acid metabolism, and energy-related metabolic regulation. The increased levels of adenosine, L-serine, and L-methionine, together with the differential expression of purine-metabolic genes, reflect changes in nucleotide turnover and amino acid utilization in the high-altitude population. Purine metabolism is closely linked to ATP turnover and oxygen-related metabolic responses, while serine- and methionine-associated pathways are involved in nucleotide synthesis and redox regulation.

## 5. Conclusions

This study provides an integrated assessment of altitude-associated physiological, structural, transcriptomic, and metabolomic responses in the freshwater snail *C. cathayensis*. By combining biochemical analyses, shell characterization, and multi-omics approaches, we revealed that populations inhabiting contrasting altitude-associated environments exhibited coordinated changes in antioxidant defense, energy metabolism, shell biomineralization, cellular regulation, and metabolic homeostasis. These findings expand the current understanding of environmental responses in freshwater mollusks and highlight the potential roles of molecular and metabolic regulation in coping with habitat variation. However, because this study was based on comparisons between naturally occurring populations, the observed differences may result from a combination of genetic differentiation and phenotypic plasticity. The relative contributions of these mechanisms cannot be distinguished based on the current data alone. Future studies using common garden experiments, reciprocal transplantation, and controlled environmental manipulations will be necessary to further clarify the mechanisms underlying altitude associated responses in *C. cathayensis*.

## Figures and Tables

**Table 1:** Significantly different metabolites.

Metabolic Pathway (KEGG Pathway)	Metabolite	ESI+/−	Rt (min)	*m*/*z*	VIP Value	*p*-Value	Highlan vs. Lowland
Cysteine and methionine metabolism	L-Serine	ESI−	0.8641	104.0350	1.0551	0.0001596	UP
	Sulfate	ESI−	1.1581	96.9600	1.0395	0.0005621	UP
	L-Methionine	ESI−	1.0314	148.0430	1.8825	0.006212	Up
Circadian entrainment	Melatonin	ESI−	0.9826	291.1341	2.1481	0.00334	Down
	Dg(11D5/11D3/0:0)	ESI−	8.4914	709.5398	1.3784	0.004766	Down
Steroid hormone biosynthesis	Prasterone	ESI+	6.7841	289.2137	1.9799	0.004697	Down
	Tetrahydrodeoxycorticosterone	ESI+	7.5344	335.2583	1.7593	0.00001401	Down
	Tetrahydrocorticosterone	ESI−	7.1468	349.2352	1.4708	0.0385	Down
	Dehydroepiandrosterone Sulfate	ESI−	0.8341	367.1559	1.0136	0.005726	Up
ABC transporters	Adenosine	ESI+	0.9196	268.1017	1.5323	0.01376	UP
	Riboflavin	ESI+	2.9782	399.1239	1.8092	0.01083	UP
Biosynthesis of unsaturated fatty acids	11,14-Eicosadienoic Acid	ESI+	8.0457	363.2896	1.8404	0.03309	Down
	Dihomo-Alpha-Linolenic Acid	ESI+	8.2985	339.2871	2.5519	0.0001563	Down
	Ganosporeric Acid A	ESI+	7.6122	549.2443	1.5643	0.02272	Down
	13,16-Docosadienoic Acid	ESI−	9.3487	381.2982	1.1375	0.02664	up

## Data Availability

The genome assembly data are available in the Genome Sequence Archive (GSA) at CNCB under Project No. PRJCA066856. The transcriptome sequencing data are available in the NCBI Sequence Read Archive (SRA) under accession number PRJNA1478776.

## Supplementary Materials

The following supporting information can be downloaded at: https://www.mdpi.com/article/10.3390/ani16152440/s1, Table S1. Primers used for RT-qPCR validation of RNA-seq data. Table S2. Summary statistics of raw and clean reads from the RNA-seq libraries of *C. cathayensis*. Table S3. Differentially expressed genes (DEGs) identified between the high-altitude and low-altitude populations of *Cipangopaludina cathayensis*. Table S4. Top 20 Pearson correlations between differentially expressed genes and metabolites.
