# Supplementary material for: Altitude-Related Adaptation in Freshwater Snails (Cipangopaludina cathayensis): Insights from Biochemical, Transcriptomic, and Metabolomic Analyses
Source: Animals (Basel). 2026 Aug 6;16(15):2440. doi: 10.3390/ani16152440 (PMC13463894; doi:10.3390/ani16152440)
Supplement: Supplementary file 1 [file animals-16-02440-s001.zip › Table S1.pdf]

Table S1. Primers used for RT-qPCR validation of RNA-seq data.

Gene ID	Name	Forward primer (5'-3')	Reverse primer (5'-3')	RNA-seq log_2FC	qPCR log_2FC
ytl.chr7G51230.1	ACNAT2	TGGCGGTGTCCAGAGGTT	GCGGGATTCTGCTGACGA	1.00	1.02
ytl.chr4G39090.1	STK31	ACCCAACATTTCGCCTCC	ACCGCAATAATCAGTTCCTT	1.47	1.15
ytl.chr7G40730.1	PTPRK	ACTGCCCAATGTTCCTCT	ATAATGCTGCGGTCTTCC	1.51	1.38
ytl.chr2G26010.1	STAT5B	CCACTTCTTGGCATCTAA	CTCTTGGGCTTGTACTTT	1.34	1.29
ytl.chr2G06020.1	FUT1	AGGAGCACATCAATAGCC	GTGGGTGACAGCGTAGAA	1.25	1.90
ytl.chr8G27540.1	CCL1	CCAATGGGTTTGTTTATG	GCTACAGGGTCAGGTTTT	-0.06	-1.73
ytl.chr1G31180.1	PXYLP1	CATACGCATAGTGAGACA	AACTTTGAGATTTGGTGG	-1.80	-2.42
ytl.chr6G13980.1	ARRDC3	ACCTACACTAACACCACGAC	AAAGGGACAGAACCTATGA	-0.88	-1.08
ytl.chr8G54060.1	LRIG3	TTTTATCGGCATCACAGA	GCAAATGAACTAGCACCT	-2.38	-1.92
ytl.chr6G06550.1	SLC2A1	CAGTTTACGGTTCCAGAC	AGCATATTCAGAGGCAAG	-1.58	-1.53
