# Supplementary material for: Altitude-Related Adaptation in Freshwater Snails (Cipangopaludina cathayensis): Insights from Biochemical, Transcriptomic, and Metabolomic Analyses
Source: Animals (Basel). 2026 Aug 6;16(15):2440. doi: 10.3390/ani16152440 (PMC13463894; doi:10.3390/ani16152440)
Supplement: Supplementary file 1 [file animals-16-02440-s001.zip › Table S2.pdf]

Table S2. Summary statistics of raw and clean reads from the RNA-seq libraries of *C. cathayensis*.

Sample	RIN	Raw read	Clean read	Error rate (%)	Q20(%)	Q30(%)	GC content (%)
High1	9.90	45,426,828	45,143,058	0.0115	99.03	96.83	41.92
High2	9.90	43,841,130	43,510,096	0.0116	99.01	96.76	42.10
High3	10.00	52,215,006	51,841,030	0.0116	99.01	96.76	42.55
High4	9.40	43,392,818	43,107,532	0.0116	99.00	96.73	42.16
High5	10.00	46,666,770	46,376,882	0.0116	99.02	96.78	42.61
High6	9.70	40,339,932	40,045,716	0.0115	99.03	96.85	42.66
Low1	9.90	42,472,552	42,169,172	0.0116	98.97	96.67	41.32
Low2	9.00	44,489,322	44,128,762	0.0116	98.96	96.66	41.39
Low3	9.60	40,784,500	40,487,044	0.0116	98.96	96.66	41.82
Low4	9.40	43,277,868	42,930,806	0.0116	98.99	96.76	41.08
Low5	9.80	44,208,670	43,884,322	0.0116	99.01	96.78	41.20
Low6	9.80	44,318,808	43,916,502	0.0117	98.97	96.66	41.72
