# Supplementary material for: Altitude-Related Adaptation in Freshwater Snails (Cipangopaludina cathayensis): Insights from Biochemical, Transcriptomic, and Metabolomic Analyses
Source: Animals (Basel). 2026 Aug 6;16(15):2440. doi: 10.3390/ani16152440 (PMC13463894; doi:10.3390/ani16152440)
Supplement: Supplementary file 1 [file animals-16-02440-s001.zip › Table S4.pdf]

Table S4. Top 20 Pearson correlations between differentially expressed genes and metabolites

Rank	Gene ID	Gene Name	Gene Description	Gene log2FC (High/Low)	Metab ID	Metabolite	Metabolite log2FC (HH/PT)	Pearson R	Correlation Direction	P value	BH FDR
1	ytl.chr8G51050	IGFBP-like	Insulin-like growth factor-binding protein 1.	-3.171	metab_2299	Glyceryl Palmitoleate	-0.621	**0.987**	Positive	2.85E-09	1.77E-03
2	ytl.chr7G34470	AAEL003161	Adenylosuccinate synthetase isozyme 2-like.	-2.151	metab_2299	Glyceryl Palmitoleate	-0.621	**0.987**	Positive	3.38E-09	1.77E-03
3	ytl.chr3G09050	ANK_2a	Predicted protein containing a ANK_2a domain.	-2.451	metab_4296	Digitoxin	-1.083	**0.985**	Positive	5.65E-09	1.97E-03
4	ytl.chr4G31430	LRP2	LOW QUALITY PROTEIN: low-density lipoprotein receptor-related protein 2-like.	1.178	metab_2206	Stearoyl Lysophosphatidylethanolamine	0.252	**-0.982**	Negative	1.32E-08	2.32E-03
5	ytl.chr3G06710	ACAA2	3-ketoacyl-CoA thiolase, mitochondrial-like.	-1.069	metab_5190	N-[(Z)-(6-Methoxy-2-Oxo-1-Pentylindol-3-Ylidene)Amino]Benzamide	-0.701	**0.982**	Positive	1.42E-08	2.32E-03
6	ytl.chr8G37790	CNFN-A	Cornifelin homolog.	-1.563	metab_5032	Pc(Pgd1/P-16:0)	0.821	**-0.982**	Negative	1.43E-08	2.32E-03
7	ytl.chr4G04200	TNKS2	Putative Tankyrase-2 homolog.	1.441	metab_496	Britannilactone Diacetate	-1.579	**-0.982**	Negative	1.55E-08	2.32E-03
8	ytl.chr1G07550	Unannotated gene	No reliable functional annotation was identified in the available databases.	4.468	metab_1290	Dehydrocurdione	0.461	**0.980**	Positive	2.20E-08	2.42E-03
9	ytl.chr2G27200	CHST1	Carbohydrate sulfotransferase 1-like.	-1.471	metab_2299	Glyceryl Palmitoleate	-0.621	**0.980**	Positive	2.55E-08	2.42E-03
10	ytl.chr9G43500	mucin-5AC-like	mucin-5AC-like.	-1.409	metab_2299	Glyceryl Palmitoleate	-0.621	**0.980**	Positive	2.64E-08	2.42E-03
11	ytl.chr8G00270	DOCK7	Dedicator of cytokinesis protein 7-like.	-1.138	metab_5190	N-[(Z)-(6-Methoxy-2-Oxo-1-Pentylindol-3-Ylidene)Amino]Benzamide	-0.701	**0.980**	Positive	2.71E-08	2.42E-03
12	ytl.chr8G24120	Unannotated gene	No reliable functional annotation was identified in the available databases.	-5.181	metab_6342	Nonactin	-0.736	**0.979**	Positive	2.77E-08	2.42E-03
13	ytl.chr8G46210	FGD4	FYVE, RhoGEF and PH domain-containing protein 4-like.	3.521	metab_5033	Pe(Dime(13,5)/20:3(5Z,8Z,14Z)-O(11S,12R))	0.925	**0.979**	Positive	3.10E-08	2.50E-03
14	ytl.chr6G29650	CPT1A	carnitine O-palmitoyltransferase 1, liver isoform-like.	2.270	metab_2910	Oridonin	-0.244	**-0.978**	Negative	4.03E-08	2.61E-03
15	ytl.chr6G37280	VOC domain-containing protein	Putative VOC domain-containing protein homolog.	-2.349	metab_6330	Cynarasaponin E	-0.252	**0.977**	Positive	4.59E-08	2.61E-03
16	ytl.chr3G20040	Perlucin-like protein	Perlucin-like protein.	-2.048	metab_172	Adenosine	0.395	**-0.977**	Negative	4.62E-08	2.61E-03
17	ytl.chr8G21980	actin, cytoskeletal 1A	Putative actin, cytoskeletal 1A homolog.	2.972	metab_1340	Methyl Linolenate	-0.218	**-0.977**	Negative	5.16E-08	2.61E-03
18	ytl.chr6G05010	Ficolin-like	Ficolin-2-like.	-1.224	metab_2299	Glyceryl Palmitoleate	-0.621	**0.977**	Positive	5.27E-08	2.61E-03
19	ytl.chr7G54730	GLO1	Lactoylglutathione lyase-like.	-1.330	metab_5190	N-[(Z)-(6-Methoxy-2-Oxo-1-Pentylindol-3-Ylidene)Amino]Benzamide	-0.701	**0.977**	Positive	5.32E-08	2.61E-03
20	ytl.chr9G44570	CYP26-like	Putative cytochrome P450 4F4 homolog.	1.558	metab_2532	Dpo-1	-0.182	**-0.977**	Negative	5.41E-08	2.61E-03
